# Microbial custody: key microbiome inhabitant *Sphingomonas* alleviates silver nanoparticle toxicity in *Daphnia magna*

**DOI:** 10.1093/femsec/fiaf061

**Published:** 2025-06-06

**Authors:** Jesse Ouwehand, Bregje W Brinkmann, Willie J G M Peijnenburg, Martina G Vijver

**Affiliations:** Institute of Environmental Sciences (CML), Leiden University, PO Box 9518 Leiden 2300 RA, The Netherlands; Institute of Environmental Sciences (CML), Leiden University, PO Box 9518 Leiden 2300 RA, The Netherlands; Institute of Environmental Sciences (CML), Leiden University, PO Box 9518 Leiden 2300 RA, The Netherlands; National Institute of Public Health and the Environment (RIVM), PO Box 1 Bilthoven, The Netherlands; Institute of Environmental Sciences (CML), Leiden University, PO Box 9518 Leiden 2300 RA, The Netherlands

**Keywords:** bacterial resistance, metal speciation, metal-based nanoparticles, microbiome-aware ecotoxicology, microbiome-host

## Abstract

Increased usage of nanotechnological applications inevitably leads to exposure of hosts and their associated microbiomes to metallic nanoparticles. Various bacteria within the microbiome harbour mechanisms to protect themselves against metal-related toxicity. These mechanisms have been broadly described in the absence of a host. Here, we studied how silver ion-resistant bacteria isolated from the *Daphnia magna* microbiome shape the host's exposure to silver nanoparticles. With germfree and mono-associated neonates, the effects of these microbes on the sensitivity of *D. magna* to silver nanoparticles were studied. By using this approach, a core member of the *D. magna* microbiome *Sphingomonas yanoikuyae* was identified to be silver-resistant. Neonates mono-associated with *S. yanoikuyae* were as sensitive to silver nanoparticles as naturally colonized neonates, whereas mono-association with *Microbacterium* and germfree neonates had increased sensitivity. Silver ions are the major attribution to toxicity in germfree and *Microbacterium*-associated neonates, whereas particles contribute more to the toxicity for the naturally- and *Sphingomonas*-colonized neonates. *Sphingomonas* accumulated *in vivo* more silver ions from its local environment than the other *D. magna* bacterial isolates. The current study shows that bacteria can play a vital role in shaping the speciation of nanomaterials and thereby modifying the toxicity to hosts.

## Teaser

Bacteria protect against silver nanoparticle toxicity in *Daphnia magna* by active uptake of silver ions.

## Introduction

Research on the microbiome has become increasingly more accessible due to advancements in computational and sequencing methodologies. These advancements lead to the understanding that the microbiome modulates host fitness, adaptation, and evolution (Akbar et al. 2022). Because of this interplay, the host-associated microbiota presumably forms a part of the biological variation in responses to chemical stressors (Duperron et al. 2020). Connections between a host and its associated microbiota are often based on correlations between the relative abundance of microbial species in marker gene sequencing data and the differences in the hosts’ responses to chemical exposure. Direct causal interference of the microbiota and host endpoint is scarcely studied. Arguably, these direct causal relationships are essential for deciphering and understanding the relationship between host and their associated microbiota during exposure based on sequencing data (Zhang et al. 2019, Chi et al. 2021).

Host-associated microbial species operate as one of the central players in shaping exposure by directly interacting with chemical stressors (Collins and Patterson 2020). Microbes interact, for instance, with the metals in their local environment. Various bacteria of an animal microbiome have evolved mechanisms to sense and attenuate the toxicity induced by metals (Chandrangsu et al. 2017, Argudín et al. 2019). Common bacterial strategies to reduce the impact of metal exposure include removal by efflux pumps and the sequestration and/or reduction of ionic species by inter- and extracellular metalloproteins (Robinson et al. 2001, Nies 2003). These mechanisms alter the speciation of the accumulated metal in the host (Duan et al. 2020). Metallic nanoparticles pose an ever-increasing threat to natural systems due to anthropogenic production, resulting in more intense environmental emissions. Analogous to metal ion-induced toxicity, metallic nanoparticles cause damage in the host by inducing inflammation, genotoxicity, and cellular dysfunction (Medici et al. 2021). Metallic nanoparticles need to be considered more adversely than ionic metals, as their induced toxicity emanates from the particles and the ions shedding from them (Vijver et al. 2018).

The proclaimed microbial ionic metallic resistance mechanisms could shape exposure of the host during exposure to metallic nanomaterials, especially in the case of soluble nanoparticles such as silver (Loza et al. 2014). The present study explores this relationship between microbial resistance to silver and the survival of *Daphnia magna* to silver nanoparticles. *Daphnia magna* is a well-established animal model in ecotoxicology and has become an increasingly prominent model in microbial ecology over the last decade (Ebert 2022). The *D. magna* model poses several advantages for studying bacterial function *in vivo* because *D. magna* reproduces parthenogenetically, in consequence reducing genotypic variation interfering with the microbiome (Macke et al. 2017). *Daphnia magna* holds a microbiome with relatively low taxonomic diversity compared to other commonly used model organisms while maintaining similar fundamental host-microbe interactions (Cooper and Cressler 2020, Akbar et al. 2022); *Daphnia* can survive axenically (Callens et al. 2016), and could be analysed with standardized methodology due to the vast usage in ecotoxicological testing (OECD 2004).

Altogether, the present study isolated and identified silver ion-resistant bacteria originating from the *D. magna* microbiome. Mono-associated neonates were used to study the effect of specific microbial species from the microbiome, thereby seeking if *ex vivo* resistance resolves into *in vivo* protection. Broadly, the current study works as a proof of concept that this approach can be applied to isolate and characterize microbes harbouring functions that shape host outcomes during exposure to nanomaterials.

## Material and methods

### *Daphnia magna* and bacterial collection

*Daphnia magna* was maintained in-house at 22 ± 1°C with a 16 h:8 h light: dark cycle and fed with dried *Chlorella* powder *ad libitum*. Bacteria were isolated by placing 1 neonate in a sterile Safe-Lock Tube with six Zirconium Oxide Beads and washed thrice with 350 µl sterile M7 (OECD 2004). The neonates were placed on ice for 5 min before lysing them using TissueLyzer II (Qiagen, Hilden, Germany) for 45 s at 30 Hz. Tubes were centrifuged for 10 s at max speed to collect everything at the bottom of the tube. Solutions were resuspended by pipetting and 50 µl was plated on LB agar. Plates were incubated at 22°C for 5 days. Species were isolated by plating three consecutive times on LB-agar. Species were identified based on 16S rRNA sequences, which were amplified using colony PCR reactions performed with 49 µl reaction mixture (per reaction: 5 µl 10× dreamTaq buffer, 5 µl 2 mM dNTPs, 0.5 µl 100 µM 27F-primer and 0.5 µl 100 µM 1492R-primer, 0.1 µl 5 U µL^−1^ dreamTaq polymerase, 37.9 µl nuclease-free water) and 1 µl bacterial suspension. Primer sequences used were 5′-AGAGTTTGATCMTGGCTCAG for 27F and 5′-TACGGYTACCTTGTTACGACTT for 1492R (Lane 1991). The 16S rRNA sequences were amplified using a thermocycler (Biorad, Hercules, CA) which was programmed as: initial denaturation at 95°C for 5 min; 25 cycles of 30 s at 95°C, 30 s at 66°C, and 30 s at 72°C; final step at 72°C for 10 min. Five microlitre of PCR product was examined using electrophoresis on a 1% agarose gel containing Sybr. Safe in 0.5× TBE at 120 V for 20 min. Vials with clear products were sent off to Macrogen (Amsterdam, The Netherlands) for Sanger sequencing. Sequences were identified using the Silva SSU r138.2 database and NCBI BLAST (Camacho et al. 2009, Glockner et al. 2017).

### Testing materials and characterization

Before every experiment, a fresh nanoparticle suspension was prepared by mixing 15 µl NM300K (10% w/w, RAS AG, Regensburg, Germany) in 15 ml Elendt M7 medium (OECD 2004). The particle suspension was stabilized by sonication for 10 min (Sonicor, Wallingford, CT). The hydrodynamic size (HDS) and zeta-potential were measured using the multiangle DLS Zetasizer Ultra (Malvern, Malvern, UK). A suspension of 50 µg/l was used for the measurement of the HDS and the zeta-potential and the measurements were performed after 0, 24, and 48 h. Particle size and shape were determined by transmission electron microscopy (TEM). Two microlitre of 50 µg l^−1^ suspension was applied on a 200-mesh carbon-coated copper TEM grid and dried at room temperature for 24 h. Images were made using a 100 kV JEOL1010 transmission electron microscope (Tokyo, Japan). The size was determined by measuring 60 particles in Fiji/ImageJ version 1.54f (Schindelin et al. 2012, Schneider et al. 2012).

### Sterilization and bacterial colonization of *Daphnia magna*

Germfree daphnids were obtained following the methodology reported by Callens et al. (2016) with slight modifications. Briefly, eggs older than 12 hpf still in the chorion were excised from adult daphnids using tweezers under a stereomicroscope and collected in a 15 ml flacon. The eggs were sterilized in 10 ml 0.01% peracetic acid in filter-sterilized M7 for 10 min and washed thrice with sterile M7 medium. Eggs were hatched for 48 h in 3 ml sterile M7 at 22°C. For mono-association, freshly cultured bacteria were added directly to the eggs to reach a concentration of 10^5^ CFU ml^−1^. Sterilization and colonization were verified by placing a neonate in sterile Safe-Lock Tubes with 350 µl sterile M7 and six Zirconium Oxide Beads. The neonates were placed on ice for 5 min before lysing them using a TissueLyzer II (Qiagen, Hilden, Germany) for 45 s at 30 Hz. Tubes were centrifuged for 10 s at max speed and 50 µl of suspension was plated on LB agar and plates were incubated at 22°C. No growth/contamination after 5 days was considered to be germfree.

### Acute toxicity testing

*Daphnia magna* neonates aged younger than 24 h either from the general culture or hatched *ex vivo* were selected for acute toxicity testing. Nominal exposure concentrations of 5, 10, 20, 30, and 50 µg l^−1^ NM300K in filter-sterilized M7 medium were used to determine the dose-response curve. A dose-response curve for soluble Ag was determined using a two-fold dilution series of AgNO_3_ starting from 10 µg l^−1^ to 0.625 µg l^−1^. Using these dose-response relationships, the relative contribution of particles and ions to the toxicity of the nanoparticle suspensions was calculated through the response additivity model


\begin{eqnarray*}
\overline {{{E}_{{total}}}} = 1 - \left[ {\left( {1 - \overline {{{E}_{{particle}}}} } \right)\left( {1 - \overline {{{E}_{ion}}} } \right)} \right]
\end{eqnarray*}

Using the response additivity model, an EC_50_-value was determined for the particle and ion contributions separately. Next, the relative contributions of ions and particles were determined in two steps. Firstly, the measured concentrations of particles and ions were converted to toxic units by dividing these values by the respective EC50 values from the additivity model. Secondly, the relative contributions were calculated as the ratio between the toxic units for particles or ions and the sum of both (amongst Song et al. (2023)).

Exposures were performed in sterile 12-well plates (Gibco) with 5 neonates in 2.5 ml to prevent contamination with 20 neonates per condition. This setup did not result in different results compared to the OECD 202 guidelines for colonized neonates (OECD 2004). Mortality was scored as immobility and assessed after 24 and 48 h by agitating the plate and waiting for 15 s. Sterilized and mono-associated neonates were lysed and plated after each test to check for contamination.

The total amount of silver was determined by acidifying 3 ml of suspension in a 1:1 ratio with 1% HCl and 2% HNO_3_, whereafter the samples were stored in a fridge until further analysis. For the determination of the concentration of ions in suspension, 4 ml of a suspension was centrifuged at 14 600 RCF for 30 min. Three millilitre of the supernatant was acidified in a 1:1 ratio with 1% HCl and 2% HNO_3_ and stored at 4°C. The silver concentration was measured using inductively coupled plasma mass spectrometry (ICP-MS). The relative contribution of ions and particles was determined by converting the measured concentration to toxic units based on the EC_50_ of ions and particles as determined separately.

### Bacterial silver uptake

The bacterial response to silver was analysed using silver-loaded LB-agar plates. A solution of 100 g l^−1^ AgNO_3_ in distilled water was autoclaved separately from the LB-agar. Silver was added to agar before pouring plates and allowed to solidify. A 10-times bacterial dilution was spotted on the agar plates with each spot being 10 µl suspension. Plates were incubated for 7 days at 22°C. After incubation, a colony was resuspended in 500 µl M7 in preweighed dried microcentrifuge tubes. Tubes were centrifuged for 5 min at 14 600 RCF and the supernatant was discarded. Bacterial pellets were dried for 24 h at 70°C and weighed to determine the bacterial dry weight. Pellets were digested overnight using 400 µl 0.76× aqua regia (1:3 of HNO_3_:HCl). After digestion, samples were diluted to reach an HCl concentration of 2.5% and stored at 4°C. Silver concentrations were determined using ICP-MS.

### Statistical analysis

All analyses were performed using R Statistical Software version 4.3.3 (R Core Team 2021). Dose-response models were generated with the R package *drc* using the *drm* function with the LL.4 model (Ritz et al. 2015). The lower limit was set at zero and the upper limit was set to one. EC_50_-values were compared using the *compParm* function with overdispersion set to TRUE. Values were shown as mean ± standard error of triplicate measurements unless stated otherwise. Multiple comparisons were analysed using the ANCOVA model. A *P*-value lower than .05 is considered to be statistically significant.

## Results

### Particle characterization

**Figure 1.**
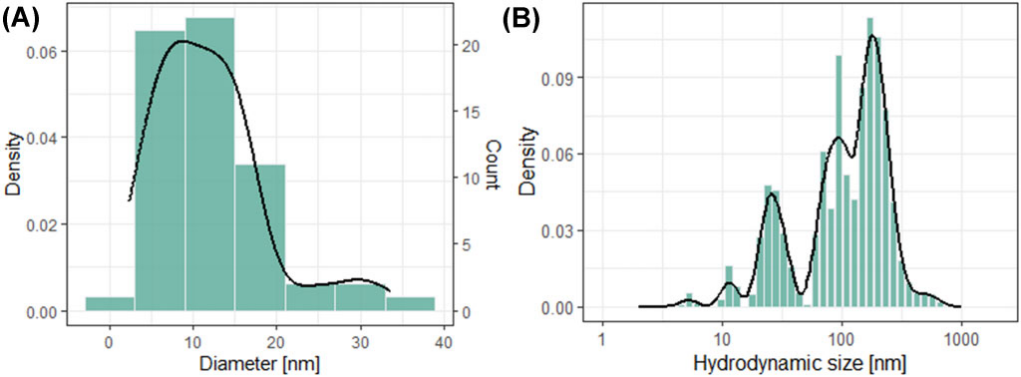
(A) The size of the particles was measured using TEM and the distribution was plotted (*n* = 60). (B) Hydrodynamic size was determined using DLS.

Silver nanoparticles of the series NM300K are small spherical particles with a mean size of 11 ± 1 nm (*n* = 60, Fig. 1A). The particles have a polydispersity index of 0.61 ± 0.07 and a zeta-potential of −20 ± 1 mV in Elendt M7 medium. The initial HDS distribution is multimodal, indicating the presence of differently sized agglomerates (Fig. 1B). The majority of the agglomerates surround the 50 nm and 180 nm as observed by a high peak density. Over the course of 24 h, the distribution becomes monomodal surrounding the 180 nm. The HDS distribution stays monomodal at around 180 nm after 48 h (Fig. S1).

### Silver-resistant bacteria from the *D. magna* microbiome

**Figure 2.**
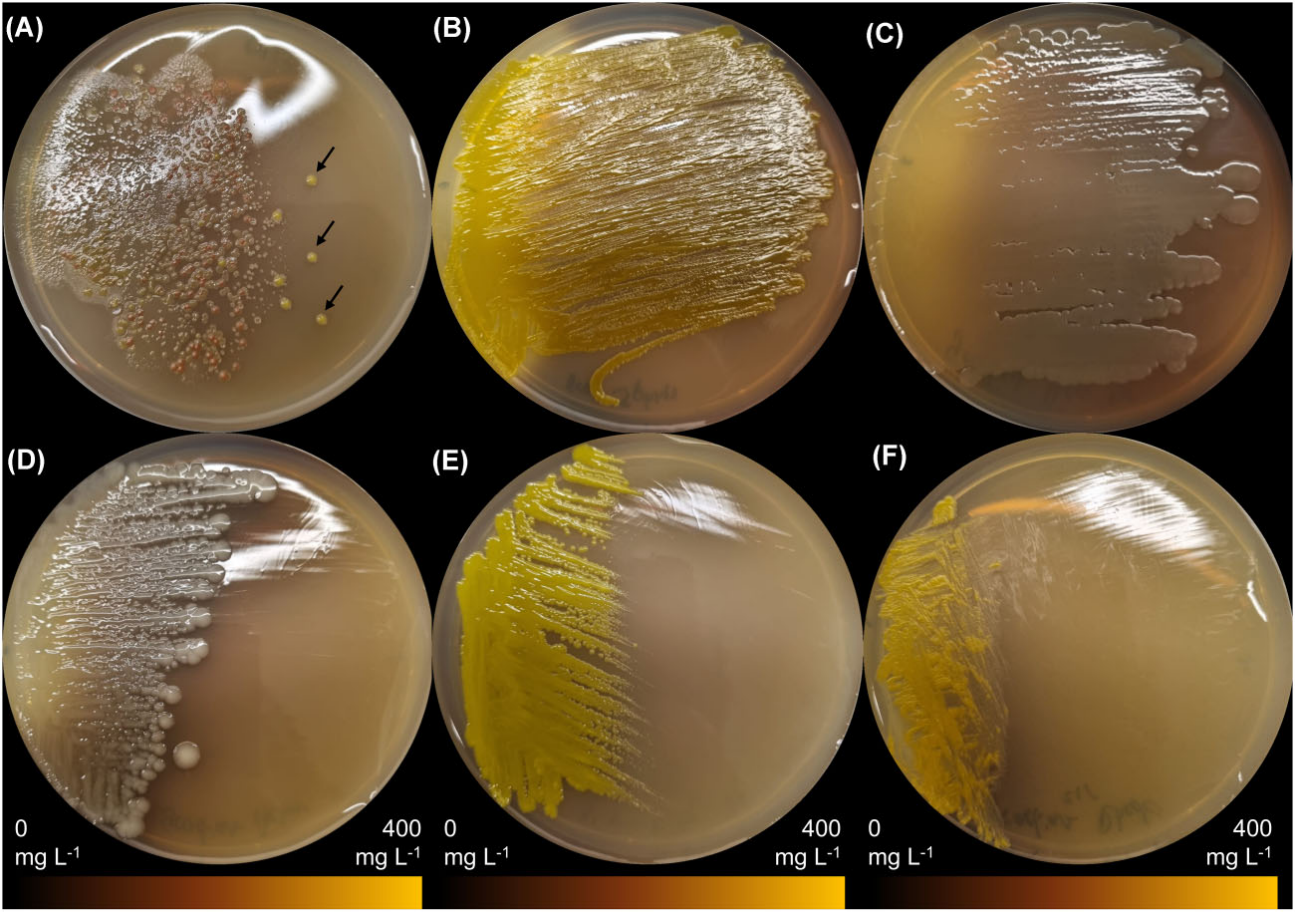
Silver nitrate gradient plates inoculated with isolated strains from *D. magna*. (A) Full *D. magna* microbiota. Arrows indicate isolates with high resilience. (B) *Sphingomonas yanoikuyae* (arrowed isolate). (C) *Aeromonas rivipollensis*. (D) *Acinetobacter johnsonii*. (E) *Microbacterium* sp. (F) *Rhodococcus* sp.

Gradient plates with silver nitrate were used to assess the resistance of the isolated bacteria from the host-related microbiota of *D. magna* to silver ions. The microbiota has a clear range of species with a distinct resistance to silver (Fig. 2A). One particular microbial isolate is prominently more resistant than other culturable microbes from the *Daphnia* microbiota. Sequencing of the 16S rRNA demonstrated that this isolate belongs to the species *Sphingomonas yanoikuyae* (refer to Table S1 for accession numbers). Spreading this isolate on a separate gradient plate showed high resistance to silver over the whole concentration range. The resistance of this species to silver was further compared to that of four other clonal isolates, *Aeromonas rivipollensis* (Fig. 2C); *Acinetobacter johnsonii* (Fig. 2D); *Microbacterium* sp. (Fig. 2E); *Rhodococcus* sp. (Fig. 2F). This showed that *Aeromonas* has a comparable resistance to silver as *S. yanoikuyae* on gradient plates. In contrast, the other isolated bacteria show higher susceptibility to silver than *Sphingomonas* and *Aeromonas*.

### *Sphingomonas yanoikuyae* protects *D. magna* against silver nanoparticles

**Figure 3.**
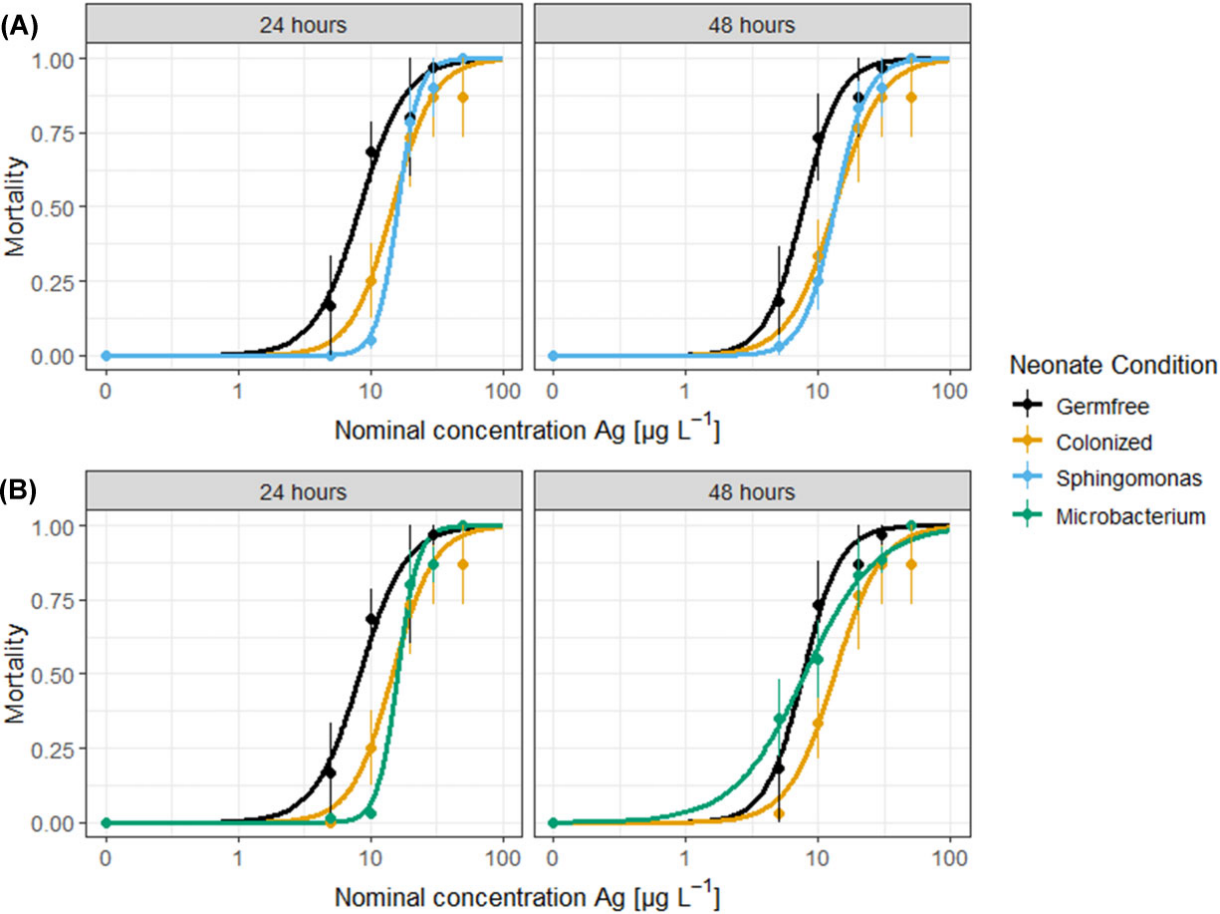
Dose response of germfree, colonized and either mono-association with *Sphingomonas yanoikuyae* (A) or *Microbacterium* (B) at 24 and 48 h after exposure. Error bars depict standard error (*n* = 3).

*Sphingomonas yanoikuyae* displayed signs of silver resistance. Therefore, its effect on silver nanoparticle toxicity to *D. magna* was examined as a follow-up. To study this effect, mono-associations of *D. magna* with silver ion-resistant *Sphingomonas* isolate and silver ion-susceptible *Microbacterium* isolate were prepared. The sensitivity of these mono-associated neonates to silver nanoparticles was compared to that of axenic and naturally colonized neonates. Neonates that were mono-associated with *Microbacterium* harboured 4.7105 ± 0.6 CFU neonate^−1^ and neonates with *S. yanoikuyae* compromised 6.0105 ± 1.0 CFU neonate^−1^, indicating an equal initial number of bacteria for both mono-associations (*P* = .12). The EC_50_-values of axenic and naturally colonized *D. magna* differed significantly, averaging 8.3 ± 0.6 µg l^−1^ and 14.8 ± 1 µg l^−1^ respectively at 24 h (*P* = .0004) and 7.7 ± 0.5 µg l^−1^ and 13.4 ± 1 µg L^−1^ at 48 h (*P* = .0007). *Daphnia magna* harbouring only *S. yanoikuyae* had similar EC_50_-values as a colonized *D. magna* (*P* = .99; at 48 h) and differed significantly from axenic neonates (*P* = .0002; at 48 h) with EC_50_-values of 16.1 ± 0.6 µg l^−1^ at 24 h and 13.4 ± 0.5 µg l^−1^ after 48 h of exposure (Fig. 3A). Mono-associations with *Microbacterium* sp. were equally sensitive to silver nanoparticles as colonized neonates at 24 h, given their EC_50_-values being 16.1 ± 0.8 µg l^−1^ (*P* = .515; at 24 h). However, at 48 h, their sensitivity did not differ from axenic neonates given their EC_50_-value of 7.9 ± 0.7 µg l^−1^ (*P* = .85; at 48 h, Fig. 3B).

**Figure 4.**
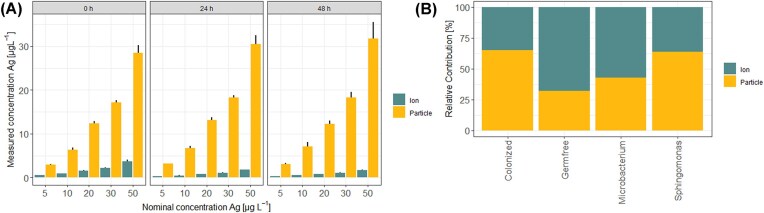
The actual concentration of particles and ions was measured using ICP-MS (A). The relative contribution of ion and particle to toxicity at EC_50_ was determined using the measured concentration using ICP-MS and the determined dose-response curves (B).

Silver nanoparticles dissolve in water and their toxicity is determined by the toxicity of the ions and the particles. The amount of silver ions remained stable in the exposure medium over the course of exposure (Fig. 4A). The majority of the toxicity to naturally colonized neonates at the EC_50_ (65%) is due to the contribution of the particles. Silver nanoparticle toxicity in axenic neonates is mainly attributed to the ions (68%) rather than to the particle fraction at the EC_50_. Mono-associations with *S. yanoikuyae* had a similar contribution pattern as naturally colonized neonates with the majority of the toxicity being due to the particles (64%) at the EC_50_. Mono-association with *Microbacterium* sp. slightly reduced the contribution of the ionic part, yet the majority of the toxicity could still be attributed to the ions (57%) at the EC_50_ (Fig. 4B).

### *Sphingomonas yanoikuyae* sequesters silver

**Figure 5.**
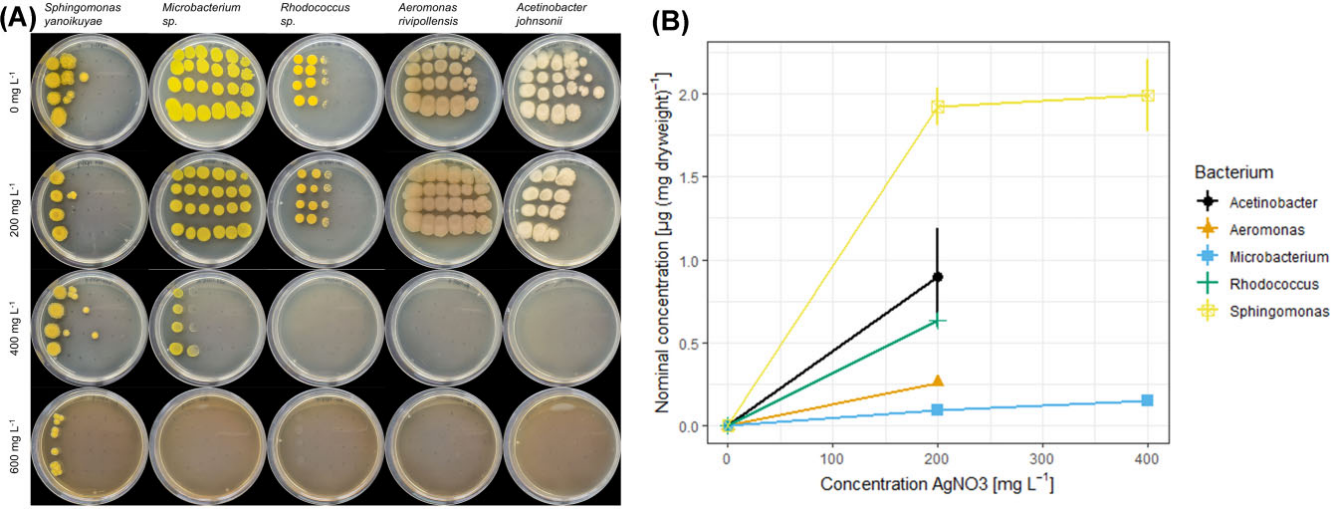
(A) LB-agar plates containing silver nitrate were inoculated with a bacterial dilution series. (B) Measured presence of silver in the diverse bacterial colonies. Graph shows mean with SEM (*n* = 3).

The reduced ion-related toxicity following colonization by *S . yanoikuyae* suggests a direct interaction between silver ions and this bacterium. Spot assays were prepared to determine silver presence to further delineate the role of *S. yanoikuyae* in modulating silver dynamics. *Sphingomonas*, once again, endured the highest silver concentration compared with other species (Fig. 5A). Only at the highest bacterial dilutions, *Microbacterium* could survive 400 mg l^−1^ AgNO_3_, indicating that abundance partially drives the tolerance to silver ions. *Sphingomonas* altered the particle and ionic contribution during exposure to *Daphnia magna*. This alteration could be attributed to the active uptake of silver ions by the bacterium (Fig. 5B). *Sphingomonas* accumulated more silver over 7 days exposure than the other bacterial species (*P* = .017) with an average of 1.9 ± 0.1 µg Ag per mg dry weight at 200 mg l^−1^ AgNO_3_. Only *Rhodococcus* and *Acinetobacter* seemed to accumulate silver with 0.6 ± 0.04 µg Ag per mg dry weight and 0.9 ± 0.2 µg Ag per mg dry weight, respectively. *Microbacterium* and *Aeromonas* accumulated silver quantities of around 0.09 ± 0.02 µg Ag per mg dry weight and 0.3 ± 0.02 µg Ag per mg dry weight, respectively.

## Discussion

Host-associated microbes partially shape the chemical-induced host outcome (Collins and Patterson 2020, Duperron et al. 2020). Here, we studied this process using soluble metallic nanomaterials focussing on the metal speciation and the impact on the host. The effect of silver-resistant bacteria on silver speciation was examined using mono-association with the *D. magna* model.

Akin to zebrafish, the *D. magna* microbiome offers protection against silver nanoparticles as seen by an increased susceptibility in germfree neonates (Brinkmann et al. 2020). The microbiome covers the organism's epithelia, forming an interactive barrier between the internal and external environment (Jandhyala et al. 2015). In the current study, we found that the presence of just one bacterial species could protect germfree neonates against silver-induced toxicity since mono-association with *Microbacterium* sp. delayed host mortality by 24 h. After 48 h, however, only *S. yanoikuyae* protected the host. The genus *Sphingomonas* conventionally inhabits the *Daphnia* microbiota in both laboratory and wild populations, making it reasonably a core member (Cooper and Cressler 2020, Frankel-Bricker et al. 2020, Houwenhuyse et al. 2023). *Sphingomonas* sp. are proficient in managing metals in its local environment since oxidoreductases reduce ions, metallothionein-related proteins bind and thereby sequester ions, and the efflux mechanism ensures that *Sphingomonas* survives (Asaf et al. 2020). This proficiency gets illustrated by the higher uptake of silver by *Sphingomonas* as compared to the other bacterial isolates, hence reducing the number of ions in their local environment. Empirically, the contribution of silver ions to the observed toxicity of *D. magna* is reduced when colonized by *S. yanoikuyae* alone. Based on the results reported here, *S. yanoikuyae*, reasonably, sequesters and reduces silver ions *in vivo*, resulting in the survival of the neonates.

Here, we showed an approach to isolate and identify chemical-resilient bacteria and assess their resilience *in* and *ex vivo*. Given this method, microbial interactions with the chemical stressor in question are supposedly the underlying reason for the differences in host outcome. Admittedly, further microbial secondary metabolism of the microbes can still influence the host outcome (Fischbach and Segre 2016). For instance, we found during the acute toxicity assessment that *A. rivipollensis* had a similar response as *Microbacterium* and *S. yanoikuyae* within the first 24 h yet caused utter mortality in the next 24 h (Fig. S3). This rapid demise of neonates can be attributed to both the opportunistic pathogenic nature of *Aeromonas* sp. and the absence of gut symbionts that stabilize and mitigate its actions (Hornak and Corno 2012, Goncalves Pessoa et al. 2019). Nevertheless, strain-specific effects should still be considered because another mono-association study showed that Aeromonas can also be beneficial to the host (Sison-Mangus et al. 2015).

Species belonging to the *Sphingomonadaceae* family have previously been found to interact with chemical stressors in both insects and plants. In particular, supplementing with *Sphingomonas* protects both aphids and moth larvae from insecticide-induced mortality by breaking down the compounds in question (Lv et al. 2023, Gu et al. 2024). In addition, the depletion of *Sphingomonas* from their respective microbiomes increased their susceptibility to their supplied chemical stressor. Colonizing plants with *Sphingomonas* attenuated cadmium and zinc stress by reducing the accumulation of the metals, thereby hampering the stress response (Chen et al. 2014, Wang et al. 2020, Cheng et al. 2021). In concordance with the present study, this indicates that *Sphingomonas* can modulate chemical stressors within host-associated microbial communities, thereby protecting the host.

Exposure to silver nanoparticles does not necessarily increase the relative abundance of *Sphingomonadaceae* within the microbiota of aquatic animals, indicating that resilience does not necessarily grant a growth advantage to this family (Li et al. 2022, Ni et al. 2022). The described capability to sequester and neutralize chemicals can, however, protect other microbial species within its proximity. In other terms, being proficient at attenuating stressors also supports gut inhabitants that do not exhibit these defence capabilities, thereby shaping the community dynamics (Bottery et al. 2021). This local protection aids to a certain extent with the community's robustness (Hellal et al. 2023). Further extrapolation to other microbial species is still infeasible as the bacterial resilience mechanism should be known. *Sphingomonas* can harbour a variety of silver-resistance genes which are widespread among bacterial species (Liu et al. 2020, Song et al. 2024). Hence, other microbiome members besides *Sphingomonas* could provide similar protection through active ion uptake. Irrespectively, this study exemplifies that active ion uptake by microbiome members can play a major role in protecting the host during exposure to silver nanomaterials.

## Conclusion

The microbiome plays a crucial role in modulating host interactions with nanomaterials, thereby influencing the host's physiological response. Despite its significance, the mechanisms underlying microbial-nanomaterial interplay within hosts remain poorly understood. Notably, microbiome constituents that sequester environmental ions can significantly impact host resilience, as exemplified in this study, where *Sphingomonas* influenced the survival of *Daphnia magna*. This phenomenon underscores the broader functional dynamics of microbiota in vivo, extending beyond direct host-microbe interactions to encompass microbial mediation of environmental factors affecting host health.

## Supplementary Material

fiaf061_Supplemental_File
