# Supplementary material for: Microbial custody: key microbiome inhabitant Sphingomonas alleviates silver nanoparticle toxicity in Daphnia magna
Source: FEMS Microbiol Ecol. 2025 Jun 6;101(7):fiaf061. doi: 10.1093/femsec/fiaf061 (PMC12199703; doi:10.1093/femsec/fiaf061)
Supplement: fiaf061_Supplemental_File [file fiaf061_supplemental_file.docx]

**Supplementary information for**

**Microbial custody: key microbiome inhabitant *Sphingomonas* alleviates silver nanoparticle toxicity in *Daphnia magna***

Jesse Ouwehand^1*^, Bregje W. Brinkmann^1^, Willie J.G.M. Peijnenburg^1,2^, Martina G. Vijver^1^

^*^corresponding author: [j.ouwehand@cml.leidenuniv.nl](mailto:j.ouwehand@cml.leidenuniv.nl), +31613504760

1. Institute of Environmental Sciences (CML), Leiden University, P.O. Box 9518, Leiden, 2300 RA, The Netherlands

2. National Institute of Public Health and the Environment (RIVM), P.O. Box 1, Bilthoven, The Netherlands

**Contents**

1. Supplementary Table 1 – Bacterial isolates *D. magna* microbiome
2. Supplementary Figure 1 – Further particle characterisation
3. Supplementary Figure 2 – Silver ion references curves
4. Supplementary Figure 3 – *Aeromonas* dose-response curve

| Supplementary Table . Bacterial isolates D. magna microbiome | | | |  |
| --- | --- | --- | --- | --- |
| Isolate ID | Isolated from | Genus | species | Accession number Genbank |
| NDMi1 | Neonate | *Aeromonas* | *rivipollensis* | PV174593 |
| NDMi9 | Neonate | *Sphingomonas* | *yanoikuyae* | PV174594 |
| NDMi14 | Neonate | *Microbacterium* |  | PV174595 |
| NDMi15 | Neonate | *Rhodococcus* |  | PV174596 |
| NDMi32 | Neonate | *Acinetobacter* | *johnsonii* | PV174597 |


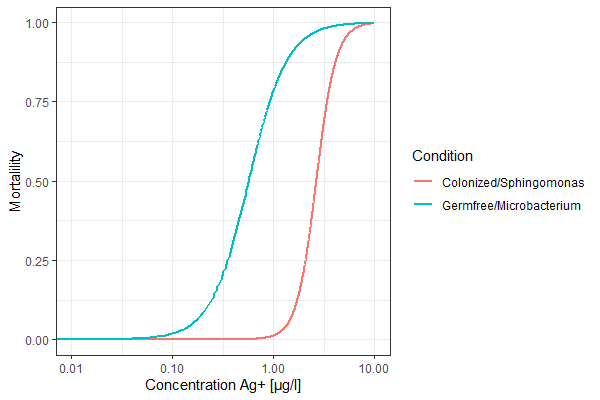


**Supplementary figure 2: Silver ion reference curve**

Reference curves for calculation of relative contribution. Due to similarity in response germfree curve is used for *Microbacterium* and colonized curved is also used for *Sphingomonas*


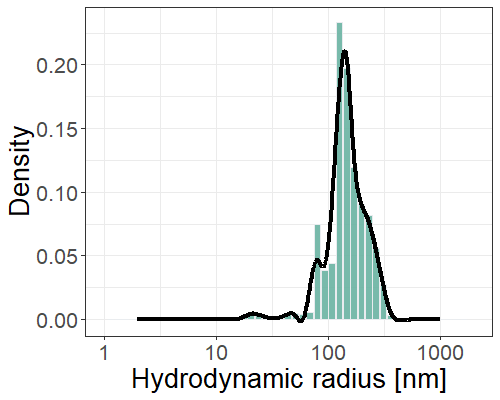

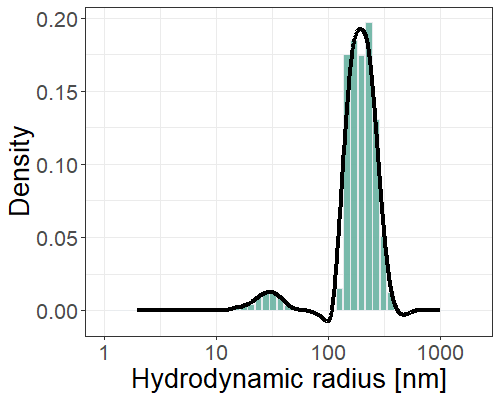


A

B


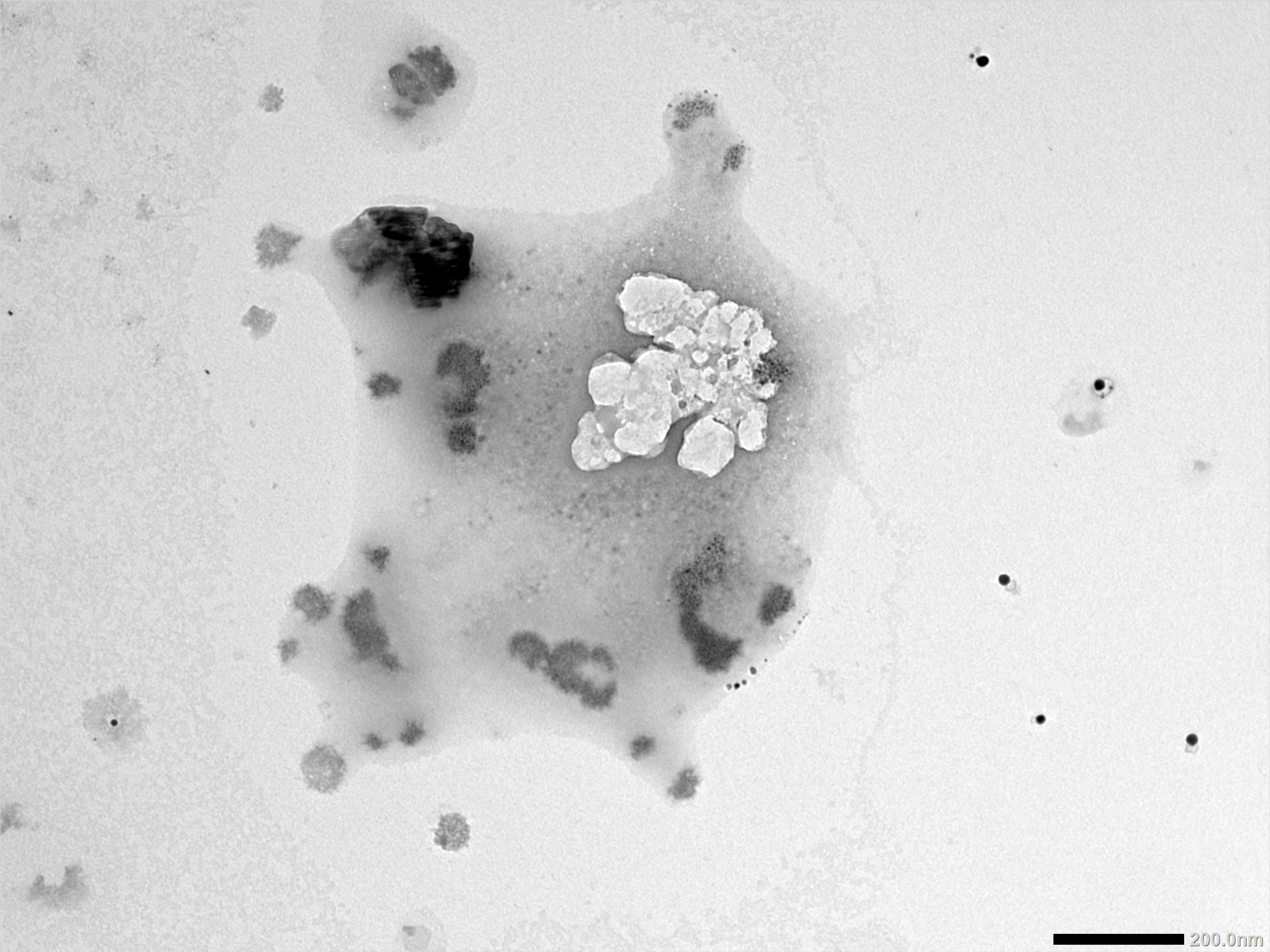


C

**Supplementary figure 1: particle information**

A: hydrodynamic radius distribution at 24h.

B: hydrodynamic radius distribution at 48h

C: TEM image of primary particles


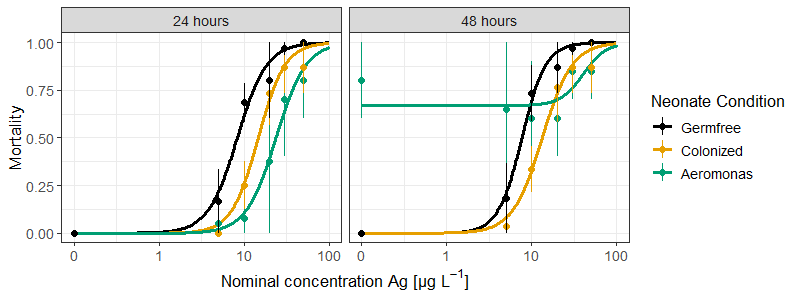


**Supplementary figure 3: Dose response of mono-associated neonates with *Aeromonas***

Dose response curves of germfree neonates, naturally-colonized, and neonates mono-associated with *Aeromonas rivipollensis* at 24 and 48 hours after silver nanoparticle exposure. Error bars depict standard error (n = 3).
